# Beyond Routine Ultrasonography: Peri-Insemination Biomarkers in Relation to Reproductive Competence in Warmblood Mares

**DOI:** 10.3390/ani16131944

**Published:** 2026-06-23

**Authors:** Raimonda Tamulionytė-Skėrė, Sigita Kerzienė, Vytuolis Žilaitis, Gintarė Vaičiulienė, Neringa Sutkevičienė, Šarūnė Sorkytė, Giedrius Palubinskas

**Affiliations:** 1 Large Animal Clinic, Veterinary Academy, Lithuanian University of Health Sciences, LT-47181 Kaunas, Lithuania; vytuolis.zilaitis@lsmu.lt; 2 Department of Animal Breeding, Lithuanian University of Health Sciences, LT-50162 Kaunas, Lithuania; sigita.kerziene@lsmu.lt (S.K.); giedrius.palubinskas@lsmu.lt (G.P.); 3 Laboratory of Animal Reproduction, Veterinary Academy, Lithuanian University of Health Sciences, LT-47181 Kaunas, Lithuania; gintare.vaiciuliene@lsmu.lt (G.V.); neringa.sutkeviciene@lsmu.lt (N.S.); sarune.sorkyte@lsmu.lt (Š.S.)

**Keywords:** estradiol, IL-6, mare, reproduction, biomarkers

## Abstract

Efficient reproductive management in mares relies on the accurate assessment of periovulatory status; however, routine ultrasonographic parameters cannot fully reflect underlying reproductive competence. The aim of this study was to evaluate hormonal (estradiol), inflammatory (IL-6), and clinical (follicle size, uterine edema, age, parity) biomarkers in relation to pregnancy outcome in mares and to assess whether these parameters may provide additional information alongside routine transrectal ultrasonographic evaluation. A total of 31 mares were examined. Estradiol concentration, follicle size, uterine edema score, and interleukin-6 (IL-6) concentration were assessed 24 h prior to insemination. Pregnancy outcomes were determined 14 days post ovulation. Statistical analysis included non-parametric tests and correlation analysis. Estradiol concentrations were significantly higher in pregnant compared to non-pregnant mares (*p* = 0.004). A moderate positive correlation was observed between mare age and estradiol concentration (Spearman’s rho = 0.599, *p* < 0.01), although age itself was not associated with pregnancy outcome. Stratified analysis confirmed higher estradiol concentrations in pregnant mares within both age groups, with statistical significance observed in younger mares (*p* = 0.003). No significant associations were found for uterine edema score, follicle size (continuous), or IL-6 concentration. These findings indicate that among the evaluated parameters, estradiol concentration at insemination showed the strongest association with subsequent pregnancy status and may provide additional information about periovulatory reproductive status. Although estradiol concentrations varied with age, its association with fertility was not solely explained by age-related differences. Rather than serving as a stand-alone predictor, peri-insemination estradiol was associated with pregnancy outcome and may provide additional information about periovulatory reproductive status. However, due to the relatively small sample size and observational design, these findings should be interpreted cautiously.

## Simple Summary

Assessment of the mare’s reproductive system competence is primarily based on ultrasonography, which remains the gold standard in clinical practice. However, successful conception depends on a range of subtle biological processes that cannot be solely visualized via transrectal ultrasonography. Therefore, there is increasing interest in identifying biomarkers that can provide additional insight into functional reproductive competence beyond structural assessment. The aim of this study was to evaluate hormonal (estradiol), inflammatory (IL-6), and clinical (follicle size, uterine edema, age, parity) biomarkers to assess whether these biomarkers provide additional insight into reproductive competence beyond routine transrectal ultrasonography evaluation of mare reproductive system. Estradiol concentrations were significantly higher in pregnant compared to non-pregnant mares (*p* = 0.004). A moderate positive correlation was observed between mare age and estradiol concentration (Spearman’s rho = 0.599, *p* < 0.01), although age itself was not associated with pregnancy outcome. No significant associations were found for uterine edema score, follicle size (continuous), or IL-6 concentration. Peri-insemination estradiol concentration was associated with pregnancy outcome and may provide additional information when evaluated together with other clinical findings.

## 1. Introduction

Efficient reproductive management in mares is essential for improving fertility rates and optimizing breeding outcomes. The equine breeding industry requires substantial economic investment, particularly when high-value semen is utilized, which underscores the importance of accurate decision-making at the time of insemination. Although ultrasonographic evaluation of the mare’s reproductive system is considered the gold standard in reproductive management, many subtle biological processes influencing successful conception occur at a level undetectable by imaging alone. Consequently, there is an increasing need to identify biomarkers that can provide additional insight into reproductive competence beyond what is possible with routine ultrasonographic examination.

Current reproductive evaluation in mares relies primarily on the ultrasonographic assessment of follicular development and uterine changes; however, these parameters mainly reflect structural features and do not fully capture the underlying biological processes necessary for successful conception [1,2]. Notably, mares may exhibit normal ultrasonographic findings during estrus, such as appropriate follicular size and uterine edema, yet still fail to conceive. This observation indicates that morphological assessment alone is insufficient to determine functional reproductive competence [3,4].

Specifically, follicular size and uterine edema indicate estrogenic stimulation but do not necessarily reflect the quality of follicular function, oocyte developmental potential, or endometrial readiness to support early embryo development [1]. In this context, there is growing interest in identifying biomarkers that can provide insight into the functional status of the reproductive system at the time of insemination [5,6].

Estradiol, produced by granulosa cells of the dominant follicle, reflects active steroidogenesis and is associated with follicular maturity and periovulatory endocrine status [7]. In addition to its role as a circulating hormone, estradiol serves as a key regulator of follicular function and intraovarian signaling. Granulosa cell activity and estradiol production are closely linked to follicular viability, vascularization, and metabolic activity, all of which contribute to oocyte developmental competence [3,8,9]. Adequate estradiol exposure during the periovulatory period has been associated with improved oocyte maturation and early embryo developmental potential [10,11]. Estradiol also plays a central role in preparing the uterine environment for embryo reception by regulating endometrial edema, uterine contractility, and gene expression associated with endometrial receptivity [5,12]. Estradiol-dependent processes are involved in embryo mobility and maternal recognition of pregnancy, both of which are critical for the establishment and maintenance of early gestation in the mare [13,14,15]. Collectively, these findings indicate that estradiol integrates multiple key components of reproductive physiology, including follicular function, oocyte competence, and uterine readiness. Recent studies underscore the value of endocrine biomarkers in equine reproduction, suggesting that hormonal assessment can complement conventional ultrasonographic evaluation by providing additional insight into reproductive function [16].

In addition to endocrine regulation, immune-mediated processes within the uterus are essential. The peri-breeding period is characterized by a transient and physiologically regulated inflammatory response within the uterus, which is essential for the clearance of spermatozoa, seminal plasma, and potential contaminants [1]. Interleukin-6 (IL-6) is a key pro-inflammatory cytokine involved in this process and plays an important role in the equine uterine immune response. It contributes to the recruitment and activation of immune cells, particularly neutrophils, which are rapidly mobilized to the uterine lumen following insemination [17,18]. In addition, IL-6 participates in the regulation and resolution of inflammation by modulating cytokine signaling pathways and coordinating the balance between pro- and anti-inflammatory responses [19,20]. Interleukin-6 has been primarily investigated in mares at the level of the uterine environment, including uterine fluid and endometrial tissue, where it functions as a key mediator of local immune activation and inflammatory signaling pathways. At the tissue level, IL-6 expression is commonly assessed through mRNA analysis in the endometrium and is considered as a marker of cytokine-mediated immune response, particularly in the context of post-breeding inflammation and endometritis [19]. Elevated IL-6 expression in uterine tissues has been associated with the recruitment of inflammatory cells and dysregulated immune responses in mares with impaired uterine clearance or persistent inflammation [21]. In contrast, considerably fewer studies have evaluated circulating IL-6 concentrations in mares. Available evidence suggests that systemic IL-6 can be increased in pathological conditions such as endometritis, indicating a link between uterine inflammation and systemic inflammatory response [22].

Pregnancy outcome in mares should be interpreted as the result of a multifactorial process involving not only mare-related factors, such as age, uterine environment, endocrine status, inflammatory response, and timing of insemination, but also stallion-related factors, including semen quality, sperm functionality, fertility potential, and semen processing conditions. Therefore, endocrine and inflammatory biomarkers evaluated in the mare should be considered complementary indicators rather than isolated predictors of conception success [23,24].

To our knowledge, comparing routine ultrasonographic findings with concurrent endocrine and inflammatory biomarkers at the time of insemination in Warmblood mares remains limited, particularly with respect to identifying biologically informative markers of reproductive competence rather than relying solely on structural assessment.

## 2. Materials and Methods

### 2.1. Ethical Approval

All procedures were conducted in accordance with Directive 2010/63/EU and were approved by the Lithuanian University of Health Sciences Bioethics Committee (No. 2025-BEC3-T-065). Informed owner consent was obtained prior to inclusion of the animals in the study.

### 2.2. Study Design

Thirty-one Warmblood mares, aged 3–21 years old, median 500 kg of weight, BCS 5–6/9 (Henneke Body Condition Scoring System) were artificially inseminated at the Lithuanian University of Health Sciences, Large Animal Clinic between March of 2025. All mares were maintained under identical management conditions, receiving the same diet consisting of hay provided at 1.5% of body weight (dry matter basis), divided into three daily feedings, with ad libitum access to water. Additionally, all mares were subjected to a standardized exercise regimen consisting of 30 min of walking per day.

All mares were evaluated prior to the study (one cycle) by ultrasonographic examination and full clinical history of parity was collected. Mares with uterine pathological signs such as endometrial cysts, uterine fluid accumulation, irregular cycles, and hyperedema were excluded from the study. One cycle per mare was studied.

**Figure 1:**
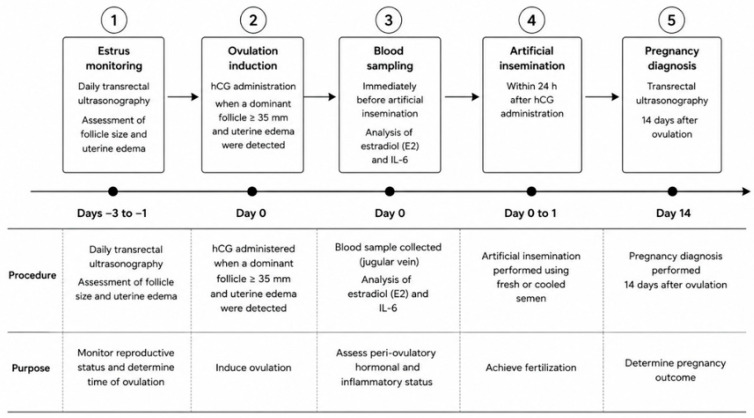
Study design and sampling timeline. Mares were monitored during estrus by transrectal ultrasonography. Ovulation was induced with hCG when a dominant follicle was ≥35 mm and uterine edema were detected. Blood samples for estradiol (E2) and IL-6 analysis were collected immediately before artificial insemination. Pregnancy diagnosis was performed by transrectal ultrasonography 14 days after ovulation.

All ultrasonographic examinations, inseminations, uterine edema scoring, and blood sampling were performed by the same experienced operator to minimize inter-observer variability (Figure 1).

### 2.3. Reproductive Management and Insemination

Mares were monitored daily during estrus using transrectal ultrasonography to assess follicular development and uterine edema.

Ultrasonographic examinations were performed using a Mindray DP-50 Vet ultrasound (Shenzhen Mindray Animal Medical Technology Co., Ltd., Shenzhen, China) equipped with a linear transrectal probe with frequency (e.g., 4.0–7.0 MHz).

The diameter of the dominant follicle was recorded in millimeters, and uterine edema was graded using a 3-point scale (1 = minimal, 2 = moderate, 3 = pronounced edema), according to [25] methodics.

Ovulation was induced when a dominant follicle ≥ 35 mm and uterine edema were detected. Ovulation was induced using human chorionic gonadotropin (hCG; 1500 IU, intramuscularly) to standardize ovulation timing across mares.

### 2.4. Artificial Insemination

Artificial insemination was performed within 24 h after hCG application.

All mares were inseminated transcervically with fresh cooled semen obtained from approved European breeding stations and transported within 24 h after collection. Semen from different stallions was used; however, to minimize stallion-related variability, only ejaculates meeting the predefined quality criteria were included. Each insemination dose contained ≥ 500 × 10^6^ progressively motile spermatozoa, with sperm concentrations typically ranging from 25 to 50 × 10^6^ spermatozoa/mL. Semen quality was evaluated before insemination.

### 2.5. Blood Collection and Laboratory Analysis

A single blood sample was collected from each mare immediately prior to insemination via jugular venipuncture into vacutainer tubes with clot activator (BD Vacutainer^®^, BD, Plymouth, UK).

Samples were centrifuged at 2460× *g* for 20 min at 4 °C. Serum was separated, aliquoted, and stored at −70 °C until analysis.

Serum concentrations of Horse E2 (Estradiol) and Horse IL6 (Interleukin 6) were determined using commercially available equine ELISA kits (ELK Biotechnology Co., Ltd., Wuhan, China), according to the manufacturer’s instructions All serum samples were analyzed in duplicate according to the manufacturer’s instructions. To minimize analytical variability, all samples were processed under the same laboratory conditions and analyzed using the same equipment and assay batches.

According to the manufacturer’s specifications, the sensitivity and detection ranges were 13.23 pg/mL and 46.88–3000 pg/mL for the Horse E2 (Estradiol) ELISA kit (Cat. No. ELK10910), and 3 pg/mL and 7.82–500 pg/mL for the Horse IL6 (Interleukin 6) ELISA kit (Cat. No. ELK5926). According to the manufacturer, the intra-assay precision CV% < 8%; inter-assay precision CV% < 10%.

### 2.6. Pregnancy Diagnosis

Pregnancy status was determined 14 days after insemination using transrectal ultrasonography based on the presence or absence of an embryonic vesicle.

For all comparative analyses, mares were classified into two groups: pregnant and non-pregnant.

### 2.7. Variables

The following variables were included in the analysis: mare age (years), parity, uterine edema score (1–3), dominant follicle size (mm), serum estradiol concentration (pg/mL), and serum interleukin-6 concentration (pg/mL).

The primary outcome variable was pregnancy status (pregnant vs. non-pregnant).

### 2.8. Statistical Analysis

Statistical analysis was performed using IBM SPSS Statistics (Version 31.0.2.0).

Data distribution was assessed using the Shapiro–Wilk test. As the data were not normally distributed and the sample size was limited, non-parametric statistical methods were applied.

Continuous variables are presented as median (minimum–maximum). Differences between pregnant and non-pregnant mares were evaluated using the Mann–Whitney U test.

For categorical analyses, estradiol concentration was divided into three groups (≤95, 96–382, and >382 pg/mL), and follicle size into three groups (≤42, 43–48, and >48 mm). These categories were established based on data distribution and biologically relevant ranges reported in previous equine reproduction studies in order to allow for an exploratory comparison of pregnancy rates between groups. Continuous variable analyses were additionally performed using non-parametric statistical methods, and the primary association between estradiol concentration and pregnancy outcome remained significant.

Associations between categorized variables and pregnancy outcome were assessed using the Fisher–Freeman–Halton exact test.

The relationship between mare age and estradiol concentration was evaluated using Spearman’s rank correlation coefficient.

To assess the potential influence of age, mares were stratified into two age groups: 3–10 years and 11–21 years. This categorization was based on previous equine reproductive studies that used approximately 10 years as a biologically relevant threshold to distinguish younger mares from older mares, as reproductive aging in mares is associated with reduced fertility and increased endometrial degenerative and inflammatory changes (DOI: 10.1016/j.anireprosci.2015.04.007; DOI: 10.1016/j.theriogenology.2018.07.013; DOI: 10.1016/j.theriogenology.2018.07.013).

All tests were two-tailed, and statistical significance was set at *p* < 0.05.

## 3. Results

### 3.1. Descriptive Statistics and Group Comparison

A total of 31 mares were included in the analysis, of which 18 (58.1%) were classified as pregnant and 13 (41.9%) as non-pregnant.

No statistically significant differences were observed between pregnant and non-pregnant mares for age, parity, uterine edema score, follicle size, or IL-6 concentration (all *p* > 0.05).

In contrast, estradiol concentration measured at the time of insemination was significantly higher in pregnant mares compared to non-pregnant mares (*p* < 0.01).

Descriptive statistics and comparisons between groups are presented in Table 1.

### 3.2. Association Between Categorized Variables and Pregnancy Outcome

A significant association between estradiol concentration and pregnancy outcome was observed (*p* < 0.05). Mares with estradiol concentrations ≤ 95 pg/mL had a markedly lower pregnancy rate (12.5%) compared to mares with concentrations of 96–382 pg/mL (73.3%) and >382 pg/mL (75.0%).

Follicle size was also significantly associated with pregnancy outcome (*p* < 0.05). The highest pregnancy rate was observed in mares with follicle sizes of 43–48 mm (75.0%), whereas both smaller (≤42 mm) and larger (>48 mm) follicles were associated with lower pregnancy rates.

No significant association was found between uterine edema score and pregnancy outcome (*p* = 0.81).

The associations between categorized variables and pregnancy outcome are presented in Table 2.

### 3.3. Distribution of Estradiol Concentrations

Estradiol concentrations at the time of insemination differed markedly between pregnant and non-pregnant mares. Pregnant mares exhibited higher estradiol concentrations, with a clear shift in distribution toward higher values compared to non-pregnant mares. In addition, greater variability was observed in the pregnant group, with several high-value observations. These differences were statistically significant (Mann–Whitney U test, *p* = 0.004).

**Figure 2:**
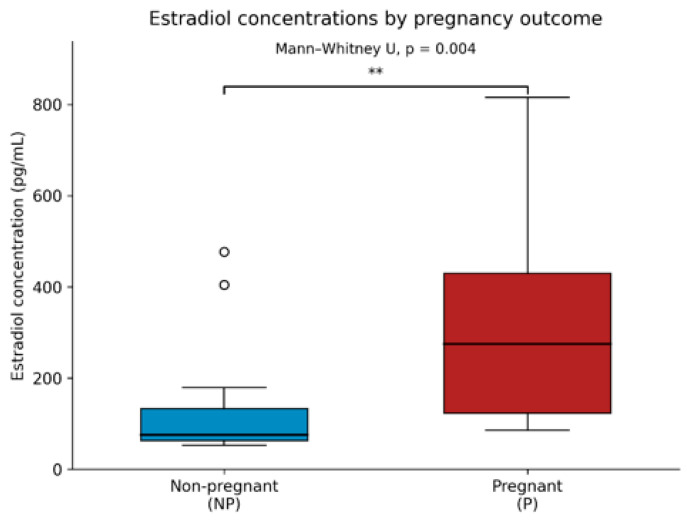
Estradiol concentrations at insemination in pregnant and non-pregnant mares. Data are presented as box-and-whisker plots showing the median, interquartile range, and distribution of values. Pregnant mares exhibited significantly higher estradiol concentrations compared to non-pregnant mares (Mann–Whitney U test, *p* = 0.004). **—*p* < 0.01.

The distribution of estradiol concentrations in pregnant and non-pregnant mares is presented in Figure 2.

### 3.4. Relationship Between Age and Estradiol Concentration

A moderate positive correlation was observed between mare age and estradiol concentration at the time of insemination (Spearman’s rho = 0.599, *p* < 0.01), indicating that estradiol concentrations tended to increase with advancing age. Despite this association, considerable variability in estradiol concentrations was observed within age groups.

**Figure 3:**
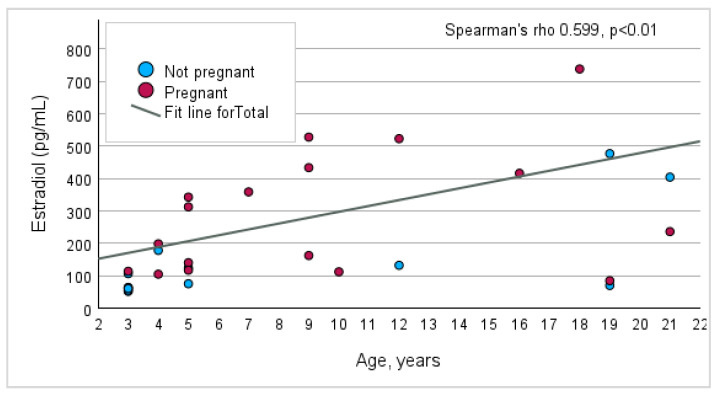
Relationship between mare age and estradiol concentration at insemination. Each point represents one mare. The line represents the overall positive association between age and estradiol concentration. A moderate positive correlation was observed (Spearman’s rho = 0.599, *p* < 0.01).

The relationship between mare age and estradiol concentration is presented in Figure 3.

### 3.5. Estradiol Concentrations Stratified by Age Group and Pregnancy Outcome

To further evaluate the potential influence of age on the relationship between estradiol concentration and pregnancy outcome, mares were stratified into two age groups (3–10 years and 11–21 years).

In mares aged 3–10 years, estradiol concentrations were significantly higher in pregnant compared to non-pregnant mares (*p* = 0.003).

In mares aged 11–21 years, a similar pattern was observed, with higher estradiol concentrations in pregnant mares; however, this difference did not reach statistical significance (*p* = 0.257).

**Figure 4:**
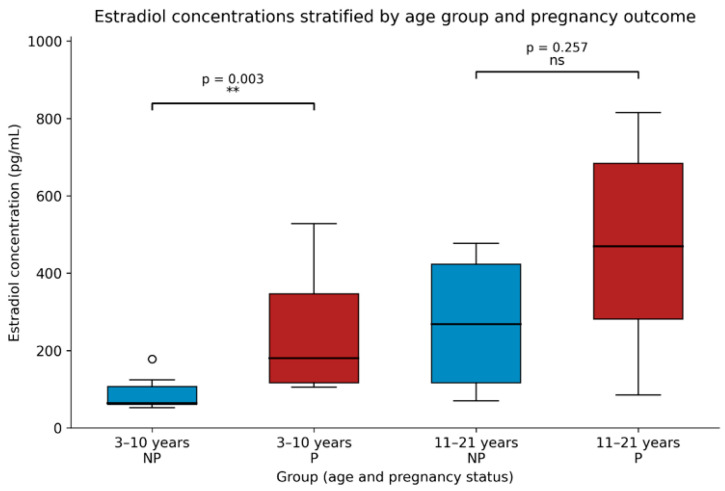
Estradiol concentrations stratified by age group (3–10 and 11–21 years) and pregnancy outcome. Pregnant mares exhibited higher estradiol concentrations within both age groups. A statistically significant difference was observed in mares aged 3–10 years (*p* = 0.003), whereas no significant difference was detected in mares aged 11–21 years (*p* = 0.257). NP—non-pregnant mares; P—pregnant mares. **—*p* < 0.01, ns—not significant.

The distribution of estradiol concentrations stratified by age group and pregnancy outcome is presented in Figure 4.

## 4. Discussion

The present findings indicate that peri-insemination estradiol offers additional insight into the functional reproductive status of mares when interpreted in conjunction with routine ultrasonographic evaluation.

Uterine edema score was not associated with pregnancy outcome, corroborating previous findings that although edema reflects estrogenic stimulation, it does not necessarily correlate with endometrial receptivity or fertility [26].

In this study, follicle size was associated with pregnancy outcome only when analyzed as a categorical variable, with the highest pregnancy rates observed in mares with intermediate follicle sizes. No differences were detected between pregnant and non-pregnant mares when follicle size was analyzed as a continuous variable. Similar findings in both classical and recent studies indicate that follicular diameter does not always accurately reflect follicular competence [10]. These results support the concept that morphological parameters alone, such as follicle size, softness, and shape, are insufficient without concurrent evaluation of biomarkers.

No association was observed between systemic IL-6 concentrations and pregnancy outcome. However, this finding should not be interpreted as evidence that IL-6 lacks biological relevance in mare reproduction. Interleukin-6 primarily acts at the local uterine level, where it regulates inflammatory responses following breeding [17,22]. Most studies investigating IL-6 in mares have focused on uterine fluid or endometrial tissue, whereas systemic cytokine concentrations may not accurately reflect the uterine microenvironment due to their low concentration, transient dynamics, and dilution in circulation. Consequently, circulating IL-6 may have limited value as a systemic biomarker of reproductive status.

To further evaluate the potential confounding effect of age, mares were stratified into two age groups. In younger mares (3–10 years), estradiol concentrations were significantly higher in pregnant mares, confirming a strong association between estradiol and pregnancy outcome in this group. In older mares (11–21 years), a similar pattern was observed, although the difference did not reach statistical significance, likely due to the smaller sample size and greater variability within this group. Despite a moderate positive correlation between age and estradiol concentration, the consistent observation of higher estradiol levels in pregnant mares across both age groups suggests that this relationship is not solely a consequence of age-related endocrine variation [10,27]. These findings indicate that estradiol concentration may be associated with periovulatory reproductive status rather than chronological age alone, supporting its potential value as an additional biomarker alongside routine clinical findings. The significantly higher estradiol concentrations observed in pregnant mares further support an association between peri-insemination endocrine status and fertility outcome [4]. Estradiol production is directly linked to granulosa cell function and follicular steroidogenesis, which are essential for oocyte maturation and the acquisition of developmental competence [28]. Additionally, adequate estradiol exposure contributes to the preparation of the uterine environment through the regulation of endometrial function and uterine contractility [1].

In this study, mares with estradiol concentrations above 95 pg/mL exhibited substantially higher pregnancy rates, suggesting a possible biological threshold that warrants further investigation. While previous studies have demonstrated associations between estradiol concentration and follicular development [27,29], clearly defined and clinically applicable thresholds for interpretation at the time of insemination remain limited. Given the considerable variability in estradiol concentrations influenced by individual, seasonal, and methodological factors, there is currently no widely accepted, clinically robust reference system for their interpretation in mares.

Overall, these findings support the concept that routine ultrasonography and circulating biomarkers provide complementary information, with ultrasonography reflecting structural status and endocrine assessment offering additional insight into functional reproductive competence.

Several limitations should be considered. The observational design of the study precludes causal inference and restricts interpretation to associations. The lack of multivariable analysis limited control over potential confounding factors, including stallion-related effects, insemination timing, and cycle-related variability. Categorization of continuous variables may have influenced the significance patterns, particularly in a relatively small dataset. These findings should be interpreted cautiously, as the categorical analyses were exploratory and may have been affected by sample size and confounding factors.

Semen from different stallions was used, which may have influenced the fertility outcomes despite the application of standardized semen quality criteria. Stallion fertility is an important determinant of pregnancy outcome in mares and could not be fully controlled. Other factors, including mare age, parity, previous reproductive performance, and seasonal influences, may also have contributed to the observed variability. Although mares were maintained under similar management conditions, the influence of these factors cannot be completely excluded.

Although only semen samples meeting the predefined quality thresholds were included, the potential influence of stallion fertility cannot be completely excluded. All biomarkers were assessed at a single time point immediately prior to insemination. Because endocrine and inflammatory changes during the periovulatory period are dynamic, a single measurement may not fully represent the biological processes associated with ovulation and early pregnancy establishment. Therefore, these results should be interpreted cautiously and as associations observed at the time of insemination rather than as a comprehensive evaluation of periovulatory dynamics. Future studies incorporating repeated sampling at different periovulatory time points would help to better characterize these changes in relation to fertility outcome.

Future studies should incorporate broader biomarker panels and assess them at multiple time points, such as before, during, and after insemination, to better capture dynamic periovulatory changes. Integration of Power Doppler ultrasonography would provide complementary real-time insight into ovarian and uterine hemodynamics, allowing for the correlation of vascular perfusion with endocrine and inflammatory biomarkers. Greater emphasis should be placed on linking systemic IL-6 and estradiol concentrations with local environments, including follicular fluid and the endometrium. Additionally, assisted reproductive technologies, such as embryo transfer and OPU-ICSI, offer valuable platforms to investigate endocrine and inflammatory regulation of follicular function and oocyte competence, particularly when combined with Doppler-based assessment of blood flow dynamics [15,30].

Peri-insemination estradiol provides relevant insight into reproductive competence, and among the evaluated parameters, demonstrated the strongest association with pregnancy outcome. Further investigation is warranted.

## 5. Conclusions

Peri-insemination estradiol concentration was the only evaluated parameter significantly associated with pregnancy outcome and may provide additional information about periovulatory reproductive status in mares. However, due to the limited sample size and observational study design, further studies are required before clinical application can be recommended. Although promising, estradiol should be interpreted within a multifactorial framework, and further studies are required to validate its clinical applicability and define robust thresholds.

## Figures and Tables

**Table 1:** Comparison of variables between pregnant and non-pregnant mares.

Variable	Not Pregnant (*n* = 13)	Pregnant (*n* = 18)	*p* Value
	Median (Minimum–Maximum)	Median (Minimum–Maximum)	
Age, years	4 (3–21)	9 (3–21)	0.125
Parity	0 (0–14)	1 (0–14)	0.211
Uterine edema score (1–3)	2 (1–3)	2 (1–3)	0.622
Follicle size, mm	46.0 (36–55)	45.5 (38–53)	0.489
Estradiol, pg/mL	75.5 (52–477)	274.5 (85–738)	<0.01
IL-6, pg/mL	4.5 (3.64–17.63)	4.85 (3.28–146.52)	0.622

Data are presented as median (minimum–maximum). *p*-values were calculated using the Mann–Whitney U test.

**Table 2:** Association of categorized periovulatory parameters with pregnancy rate in mares.

Variable	Category	n	Pregnant (%)	*p* Value
Estradiol, pg/mL	≤95	8	12.5 ^a^	<0.05
	96–382	15	73.3 ^b^	
	>382	8	75.0 ^b^	
Follicle size, mm	≤42	6	25.0 ^a^	<0.05
	43–48	18	75.0 ^b^	
	>48	7	28.6 ^ab^	
Uterine edema score	1	8	62.5	0.810
	2	13	61.5	
	3	10	50.0	

Values within the same variable with different superscript letters (^a, b^) differ significantly (*p* < 0.05). *p*-values were calculated using the Fisher–Freeman–Halton exact test.

## Data Availability

Data available on request.

## Abbreviations

The following abbreviations are used in this manuscript:

CV	Coefficient of Variation
E2	Estradiol
hCG	Human Chorionic Gonadotropin
IL-6	Interleukin-6
OPU-ICSI	Ovum Pick-Up–Intracytoplasmic Sperm Injection
